# LPS induced inflammatory responses in human peripheral blood mononuclear cells is mediated through NOX4 and G_i_α dependent PI-3kinase signalling

**DOI:** 10.1186/1476-9255-9-1

**Published:** 2012-01-12

**Authors:** Anta Ngkelo, Koremu Meja, Mike Yeadon, Ian Adcock, Paul A Kirkham

**Affiliations:** 1 Airways Disease Section, National Heart and Lung Institute, Imperial College London, London, UK; 2 University College London, Cancer Institute, London, UK; 3 Allergy and Respiratory, Pfizer, Sandwich, Kent, UK

## Abstract

COPD is a disease of innate immunity and bacterial infections are a dominant cause of exacerbations in the later stages resulting in poor health and high mortality. The pathogen-associated molecular pattern (PAMP) lipopolysaccharide (LPS) is sensed by immune cells through activation of the toll-like receptor 4 (TLR4). This leads to the activation of NADPH oxidase (NOX) and NF-κB which together drive COPD inflammation. In this study we show in human PBMCs that LPS stimulated proinflammatory cytokine release (CXCL8 and IL6) was inhibited by approximately 50% by the broad specificity phosphatidylinositol 3-kinase (PI3K) inhibitor, wortmannin. Our results also demonstrate that activation of PI3K following LPS stimulation is mediated by a NOX4 dependent mechanism releasing endogenous H_2_O_2_, as the NOX4 inhibitor apocynin blocked LPS induced AKT phosphorylation. Moreover, LPS-induced PI3K activation was inhibited by the anti-oxidant N-acetylcysteine in a concentration dependent manner (IC_50 _~100 μM). In addition, our data demonstrated that inhibition of small G proteins, by pre-treatment with pertussis toxin, inhibited LPS-induced AKT phosphorylation. Furthermore, the G-protein inhibitors pertussis toxin and mastoparan both inhibited LPS-induced CXCL8 and IL-6 release by approximately 50%. Together, these data indicate there is a mechanism in human PBMCs where TLR4 activation by LPS leads to ROS generation through NOX4 and activation of the PI3K pathway. This effect is apparently mediated through small G proteins facilitating the release of pro-inflammatory cytokines.

## Introduction

Bacterial infections are one of the dominant causes of acute exacerbations in chronic obstructive pulmonary disease (COPD). Lipopolysaccharide (LPS) is the most abundant component within the cell wall of Gram-negative bacteria. It can stimulate the release of interleukin 8 (IL-8, CXCL8, CXC ligand 8) and other inflammatory cytokines in various cell types, leading to an acute inflammatory response towards pathogens [1]. These responses are initiated by the activation of the TLR signalling through adaptor proteins, and include induction of gene expression via the activation of the NF-κB and AP-1 signal transduction pathways [2]. Bacterial LPS has been extensively used in models studying inflammation as it mimics many inflammatory effects of cytokines, such as TNF-α, IL-1β or IL-6. The cellular receptor transducing the LPS signal has been identified as Toll-like receptor 4 (TLR4) [3-5]. Binding of LPS to TLR4 leads to the activation of NF-κB through the recruitment and activation of MyD88, IL-1R kinase (IRAK), TNFR associated factor 6 (TRAF-6), as well as NADPH oxidase (Nox) [2,6,7]. NF-κB plays a crucial role in regulating the transcription of genes related to innate immunity and inflammation responses and several studies indicate its activation is controlled by reactive oxygen species (ROS) in immune modulation in the lungs and in monocytes [8-11].

Several studies searching for novel anti-inflammatory agents have led to the identification of a key role for phosphatidylinositol 3-kinase (PI3K) in transducing receptor-mediated signalling during inflammation in chronic inflammatory diseases, such as COPD [12]. The PI3K family is divided into three classes (I, II and III) depending on their structure, substrate and function [13]. The class I PI3Ks are further subdivided into class IA (p110α, p110β and p110δ) and class IB (p110γ). All class I PI3Ks mediate fundamental signalling pathways and cellular processes that orchestrate cell growth, proliferation, migration and survival [14]. Class IA PI3Ks are activated via cell surface expressed receptor tyrosine kinases (RTKs) including insulin and growth factors whereas, class IB (p110γ) are activated by G-protein coupled receptors (both α and βγ subunits of G-proteins) [15,16]. There is clear evidence that PI3Ks (PI3Kγ and PI3Kδ) play a crucial role in mediating both the innate and adaptive immune response by regulating leukocyte migration, activation and antigen response [17-19]. PI3K activation is important for neutrophil migration [20,21] and this can play a significant role in disease exacerbations where neutrophilic influx is evident, as observed in COPD.

Several studies have shown that activation of PI3Ks by many microbial stimuli such as LPS, play a key role in regulating immune cell mechanisms, such as cytokine production [22,23]. Moreover, global inhibition of the various PI3-kinase isoforms interferes with the TLR-mediated cellular signalling pathways and molecular responses such as B cell cytokine production and differentiation [24,25]. However, the molecular mechanism by which LPS induces cytokine release in human monocytes is not fully understood and therefore warrants further investigation. In this study we demonstrate that LPS-stimulated release of proinflammatory cytokines from human monocytes is mediated through the activation of PI3K in both a ROS- and a G-protein-dependent manner, propagated through NOX4 activation.

## Methods

### Isolation of human peripheral blood mononuclear cells (PBMCs) and monocytes

PBMCs from healthy volunteers were isolated by centrifugation of whole blood on Histopaque^®^-1077 (Sigma; Poole, Dorset, UK) at 400 g for 30 min at room temperature. Cells collected from the interphase were washed with PBS then resuspended in RPMI-1640 medium supplemented with 10% heat-inactivated fetal bovine serum and 2 ml L-glutamine. Human monocytes were purified by allowing the purified PBMCs resuspended in IMDM at 4 × 10^6 ^cells/ml to adhere to tissue culture plates for 1-2 hours at +37°C. Non-adherent cells were removed with warm HBSS twice before detaching adherent cells (> 90% monocytes) with cell dissociation buffer (Gibco BRL; Paisley, UK). The harvested monocytes were then resuspended in IMDM with 1% (w/v) BSA ready for the chemotaxis assay. The population of monocytes harvested by this method has been described previously [26] and the purity of the population was more than 95%. The study was approved by the local Ethics Committee with informed consent obtained from each participant.

### Cell Culture and Treatments

Purified PBMCs were pre-treated with pharmacological agents as indicated in figure legends for 30 min before stimulation with LPS (100 ng/ml) for further 15 min prior to cell lysis and assessment of pAKT/Total AKT by either MSD assay (Mesoscale; Gaithersburg, MD) or immunoblotting. Alternatively, following the addition of LPS, the PBMCs were left to incubate for 16 hours at +37°C before culture supernatants were removed and assessed for both IL-6 and CXCL8 by sandwich enzyme-linked immunosorbent assay (R&D Systems; Abingdon, UK) according to the manufacturer's protocols.

### Chemotaxis assay: (plate based assay)

Chemotaxis was assessed as previously described [27]. Briefly, monocytes were pre-loaded with Calcein-AM at 5 μg/10^7 ^cells for 20 min at +37°C, then resuspended in IMDM supplemented with 0.1% w/v BSA at 8 × 10^6 ^cells/ml and kept on ice in the dark until needed. Into the bottom well of a 96 well Neuroprobe 5 μM chemotaxis plate (Receptor Technologies Ltd; Adderbury, UK) was placed 29 μl of 3 nM C5a in medium (IMDM + 0.1% BSA), medium only (background) or cells in medium only (maximum signal). Into the upper chamber, 25 μl of calcein loaded cells were applied and the plate left to incubate for 90 min at +37°C. After 90 min unmigrated cells were washed away from the top chamber with PBS and the plate read using a Fluoroskan II plate reader (Labsystems, Cambridge, UK) in bottom reading mode (Ex 485 nm, Em 538 nm).

### Measurement of AKT/pAKT by MSD assay

Phospho (Ser473) AKT and total AKT was evaluated using the MSD^®^MULTI-SPOT assay system from Mesoscale (Gaithersburg, MD. Cat # K15100D). PBMCs were aliquoted into a 96 well tissue culture plate at 3^x^10^6 ^cells/well then incubated with or without wortmannin or N-acetylcysteine for 30 min before treating the cells with 100 ng/ml LPS for a further 15 min. Cells were pelleted at 500 g for 3 minutes at +4°C and washed once with ice cold PBS before pelleting again and lysing the cell pellet with 100 μl ice cold lysis solution provided in the MSD assay kit. 20 μg of protein cell lysate was transferred to a 96 well MSD plate and phosphor-AKT and total AKT were assessed as per manufacturer's instructions.

### Protein extraction and immunobloting

All experimental details are according to previous description (28). Whole Cell protein extracts were prepared using modified RIPA buffer (50 mM Tris HCL pH7.4. 150 mM NaCl, 1% NP-40, 0.25% Na-deoxycholate, 1% CHAPS, 1 mM EDTA with freshly added complete protease and phosphatase inhibitor cocktail II (Calbiochem; Feltham, UK). Protein concentration was determined using the Pierce BCA protein assay kit (Rockford, IL). Whole cell lysates were subjected to western blot after SDS PAGE using mouse monoclonal anti- phosphoAKT (Abcam; Cambridge, UK). Western Blots were stripped with Chemicon Re-Blot Plus western recycling kit (Chemicon International, Temecula, CA), blocked and reprobed with anti-AKT (Abcam). All other reagents were from Sigma (Poole, UK) unless otherwise stated.

### Statistical analysis

Data were analysed by means of 1-way ANOVA to determine statistically significant variance between the groups for each end point assessed. Statistical significance between groups was then calculated by using the nonparametric Mann-Whitney *t *test with GraphPad Prism software (GraphPad Software, Inc, La Jolla, Calif). Data are expressed as means ± SEMs. Differences were considered significant at a *P *value of less than 0.01.

## Results

### LPS-induced IL-6 and CXCL8 release is blocked by inhibition of PI3K

**Figure 1:**
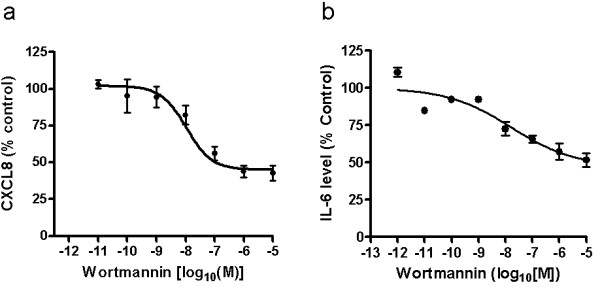
**Impact of the PI3K inhibitor wortmannin on LPS-induced release of CXCL8 and IL-6**. Purified human PBMCs were treated with 100 ng/ml LPS in the presence of wortmannin for 16 hrs. CXCL8 (a) and IL-6 (b) were assessed by ELISA and the data normalised to control (LPS stimulation only) for each experiment. The mean ± SEM for each data point from at least 3 independent experiments is shown. Sigmoidal dose response curves were plotted using Prism4 software. 100% of control (LPS-stimulated PBMCs only) represented 15 ng/ml CXCL8 and 30 ng/ml IL-6 respectively.

**Figure 2:**
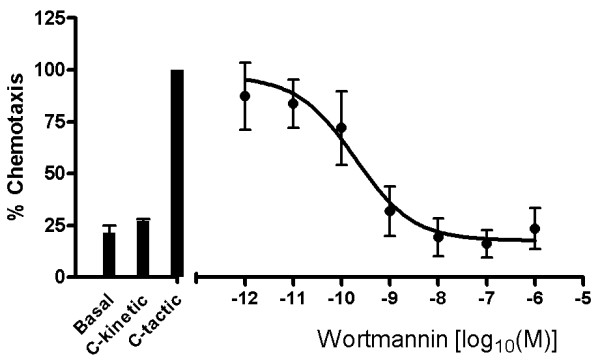
**PI3K inhibition prevents C5a-induced chemotaxis in human monocytes**. Human monocytes were pretreated with wortmannin for 30 min and then allowed to migrate in response to 3 nM C5a. Data was normalised relative to control, where control represents 100% chemotaxis in response to C5a only treatment. Results are displayed as mean ± SEM for 3 independent experiments and the sigmoidal dose response curve plotted using Prism4 software.

The impact of the general PI3K inhibitor wortmannin on CXCL8 and IL-6 release from LPS treated PBMC is displayed in Figure 1. Wortmannin inhibits both IL-6 and CXCL8 release in a concentration-dependent manner. Wortmannin inhibits the PI3kinase with an IC_50 _of 1 nM to 10 nM and did not affect affect cell viability up to a concentration of 10^-6^M. However, in both cases wortmannin at the highest concentrations used (1000 nM) was only able to inhibit approximately 50% of the total CXCL8 and IL-6 response induced by LPS. In contrast, wortmannin can be seen to completely block C5a induced monocyte chemotaxis, a PI3K-dependent functional response (Figure 2).

### LPS-induced phosphorylation of AKT is inhibited by wortmannin and N-acetylcysteine

**Figure 3:**
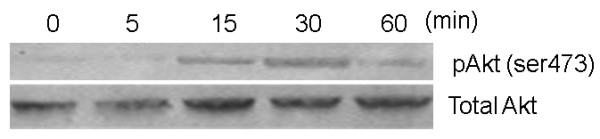
**A time course of LPS-induced phosphorylation of Akt in human PBMCs**. PBMCs were treated with 100 ng/ml LPS and at defined time points cells were harvested, lysed and assessed for the presence of Akt phosphorylation by Western blot. The blot is representative of the experiment repeated at least 3 times.

**Figure 4:**
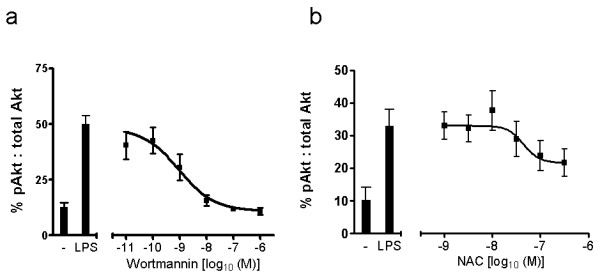
**LPS-induced phosphorylation of AKT is inhibited by wortmannin and N-acettylcysteine in a concentration-dependent manner in human PBMCs**. Human PBMCs were pre-treated with either (a) wortmanniun or (b) N-acetylcyteine for 30 minutes before stimulating with LPS for 15 minutes. For each data point, the % of phosphorylated AKT relative to total AKT present is plotted as the mean ± SEM from at least 3 independent experiments. Sigmoidal dose response curves were plotted using Prism4 software. As a negative control, cells were left untreated.

LPS stimulation of PBMCs induced phosphorylation of AKT after only 15 minutes and this was maximal by 30 minutes (Figure 3). All further experiments therefore investigated Akt phosphorylation after only 15 minutes treatment of PBMCs with LPS. Wortmannin shows a concentration dependent inhibition of LPS-induced phospho-AKT (pAKT) expression with an IC_50 _of 1 nM (Figure 4a). Inhibition of LPS-induced pAKT is complete with levels returning to those seen under basal conditions. In contrast, the anti-oxidant N-acetylcysteine was only able to inhibit LPS-induced pAKT expression by 50% at the highest concentration tested, 300 μM (Figure 4b). Nevertheless, N-acetylcysteine did show a concentration dependent inhibition of pAKT expression with an IC_50 _of around 100 μM.

### LPS induced inflammation is modulated through Gi-dependent signalling

**Figure 5:**
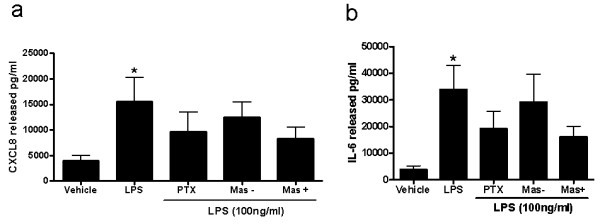
**LPS-induced release of (a) CXCL8 and (b) IL-6 in human PBMCs**. Cells were pre-treated with either the G-protein inhibitor pertussis toxin, the specific G_i_α inhibitor mastoparan (Mas+) or its inactive counterpart (Mas -). The data represents the mean ± SEM from 8 experiments. *P < 0.05 as determined by Kruskal Wallace non-parametric ANOVA followed by Dunnets post-test analysis.

The small G-protein inhibitor pertussis toxin and the specific G_i_α inhibitor 'mastoparan' inhibit G_i_α with an IC_50 _of approximately 200 pg/ml [28] and 15 μM [29] respectively. Both of these inhibitors blocked LPS-induced CXCL8 and IL-6 release by 50% (Figure 5). In contrast the negative control analogue of mastoparan failed to reduce either CXCL8 or IL-6 release from LPS-stimulated PBMCs.

### Pertussis toxin and inhibition of NADPH oxidase prevents LPS-induced phosphorylation of AKT

**Figure 6:**
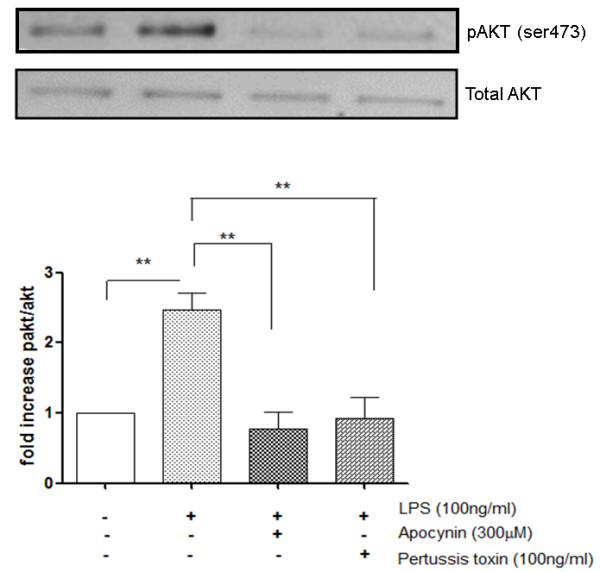
**Inhibition of LPS-induced pAkt by the G-protein inhibitor pertussis toxin and the NOX inhibitor Apocynin**. PBMCs from healthy volunteers were pretreated with either Apocynin (300 μM) or pertussis toxin (100 ng/ml) for 30 min before LPS stimulation. Phosphorylated AKT and total AKT levels were visualised by Western blot and assessed semi-quantitatively by band densitometry. The data was normalised to total AKT for each treatment group and plotted as mean ± S.E.M of 7 independent experiments (**p < 0.01 as determined by Kruskal Wallace non-parametric ANOVA followed by Dunnets post-test analysis) showing the fold-increase in phospho-Akt compared to unstimulated cells.

Pertussis toxin can completely inhibit LPS-induced phosphorylation of AKT as demonstrated by Western blotting (Figure 6). Stimulation of PBMCs with LPS induced an almost 3-fold increase in AKT phosphorylation levels compared to non-treated cells (*p *< 0.001). The compound apocynin specifically blocks NADP oxidase 4 activity with an IC50 of 10 μM [30]. The increase in AKT phosphorylation was completely inhibited by pre-treatment of the PBMCs with either pertussis toxin or the NADP oxidase 4 inhibitor, apocynin (Figure 5).

## Discussion

We report here that stimulation of human PBMC by LPS, leading to the release of pro-inflammatory cytokines such as CXCL8 and IL-6, is dependent, in part, on PI3K signalling. Moreover, the activation of PI3K by LPS was G-protein dependent as the G protein inhibitor, pertussis toxin, clearly blocked PI3K signalling. However, neither pertussis toxin nor mastoparan could completely prevent LPS induced cytokine release, indicating multiple signalling pathways were at work. In contrast to pertussis toxin, however, the anti-oxidant N-acetylcysteine could only inhibit LPS-induced PI3K signalling by 50%.

**Figure 7:**
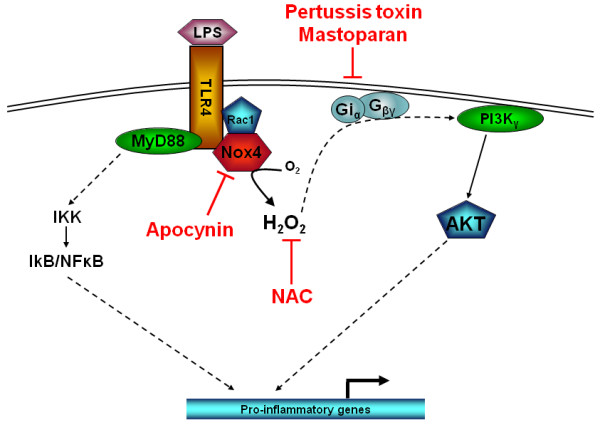
**Schematic representation of LPS-induced PI3K activity through apocynin sensitive NOX4 and pertusis toxin/mastoparan sensitive Gi protein activation**. N-acetyl cysteine neutralises any NOX4 generated intracellular superoxide which can trigger activation of ROS sensitive Gi proteins releasing G_βγ _subunits to activate PI3Kγ.

The innate immune system's ability to initiate responses to lipopolysaccharide (LPS) of Gram negative organisms is effectively carried out upon the recognition of LPS by the LPS-binding protein (LBP). LBP is an acute-phase protein that binds to LPS and catalyses its transfer to membrane-bound CD14 (mCD14), which is a 55-kDa glycoprotein expressed on the surface of monocytes in PBMCs. LPS is released from CD14 in the lipid bilayer, and the intercalated LPS binds to a complex of receptors such as the chemokine receptor 4 (CXCR4) and the heat shock proteins 70 and 90 [31]. A complex of Toll-like receptor 4 (TLR4) and MD-2 is further recruited and stimulates multiple signalling pathways such as the NF-κB. The activation of PI3K by LPS through an apparent trimeric G-protein complex would at first appear to be contradictory to current understanding, whereby only 7 transmembrane receptors are able to signal through trimeric G-protein complex activating the PI3Kγ isoform [32]. However, our results show that there is clearly a link between TLR4 receptor activation by LPS and G-protein dependent PI3K activation. A plausible explanation for this observation could be the involvement of intracellular ROS generation upon TLR4 activation by LPS. Once TLR4 is activated it recruits the NADP-oxidase complex, NOX4. This complex is then able to generate ROS in the form of hydrogen peroxide [7], which can activate PI3K [33]. The proximity of this ROS generation close to the plasma membrane would enable it to impact on other components associated with the plasma membrane. As other studies have demonstrated [34], ROS activates Gi and Go trimeric G protein complexes releasing G_βγ _subunits to activate downstream targets such as PI3Kγ. We present, therefore, a plausible mechanism to account for the G-protein dependent PI3Kγ activation as a result of LPS stimulated ROS production (Figure 7).

ROS by their very nature are highly promiscuous and will target numerous redox sensitive molecules. Indeed, the protein tyrosine phosphatases are particularly susceptible to inhibition by ROS due to a key cysteine residue within the active site [34]. This can lead to deregulated activation of PI3K and therefore increased phosphorylation of AKT [35]. When ROS was inhibited by N-acetylcysteine in our experiments, we did not see complete inhibition of PI3K activity. The explanation for this may be two fold. Firstly, not all LPS-activated PI3K is driven through ROS and secondly the N-acetylcysteine concentrations used were not as high as that reported elsewhere, where up to 30 mM N-acetylcysteine was used. Moreover, in the experiments reported by Ashebourne et al., N-acetylcysteine could not completely inhibit LPS-induced pAKT expression unlike another anti-oxidant, α-tocopherol [11]. This suggested that a compartmentalisation effect for the different anti-oxidants, whereby N-acetylcysteine, unlike tocopherol, could not penetrate the lipid compartment to neutralise the ROS located there.

In conclusion, we have shown that LPS can activate PI3Kγ activity through a ROS and pertussis toxin sensitive mechanism in human PBMC (Figure 7). Moreover, this PI3K dependent pathway is responsible for at least 50% of the functional response seen with respect to LPS-induced cytokine release from human PBMCs.

## Competing interests

The authors declare that they have no competing interests.

## Authors' contributions

AN participated in the experimental work and drafting the manuscript. KM participated in the experimental work. MY and IA participated in the study design and drafting of the manuscript. PK conceived the study, participated in its design and coordination and drafting of the manuscript. All authors read and approved the final manuscript.

## References

[B1] Sweet MJ Hume DA Endotoxin signal transduction in macrophages J Leukoc Biol 1996 60 1 8 26 8699127 10.1002/jlb.60.1.8

[B2] Akira S Toll-like receptor signaling J Biol Chem 2003 278 40 38105 8 10.1074/jbc.R300028200 12893815

[B3] Wright SD Toll, a new piece in the puzzle of innate immunity J Exp Med 1999 189 4 605 9 10.1084/jem.189.4.605 9989974 PMC2192938

[B4] Poltorak A Defective LPS signaling in C3H/HeJ and C57BL/10ScCr mice: mutations in Tlr4 gene Science 1998 282 5396 2085 8 9851930 10.1126/science.282.5396.2085

[B5] Qureshi ST Endotoxin-tolerant mice have mutations in Toll-like receptor 4 (Tlr4) J Exp Med 1999 189 4 615 25 10.1084/jem.189.4.615 9989976 PMC2192941

[B6] Akira S Takeda K Kaisho T Toll-like receptors: critical proteins linking innate and acquired immunity Nat Immunol 2001 2 8 675 80 10.1038/90609 11477402

[B7] Park HS Cutting edge: direct interaction of TLR4 with NAD(P)H oxidase 4 isozyme is essential for lipopolysaccharide-induced production of reactive oxygen species and activation of NF-kappa B J Immunol 2004 173 6 3589 93 10.4049/jimmunol.173.6.3589 15356101

[B8] Gutteridge JM Halliwell B Free radicals and antioxidants in the year 2000. A historical look to the future Ann N Y Acad Sci 2000 899 136 47 10863535 10.1111/j.1749-6632.2000.tb06182.x

[B9] Rahman I Adcock IM Oxidative stress and redox regulation of lung inflammation in COPD Eur Respir J 2006 28 1 219 42 10.1183/09031936.06.00053805 16816350

[B10] Janssen-Heininger YM Poynter ME Baeuerle PA Recent advances towards understanding redox mechanisms in the activation of nuclear factor kappaB Free Radic Biol Med 2000 28 9 1317 27 10.1016/S0891-5849(00)00218-5 10924851

[B11] Asehnoune K Involvement of reactive oxygen species in Toll-like receptor 4-dependent activation of NF-kappa B J Immunol 2004 172 4 2522 9 14764725 10.4049/jimmunol.172.4.2522

[B12] Marwick JA Chung KF Adcock IM Phosphatidylinositol 3-kinase isoforms as targets in respiratory disease Ther Adv Respir Dis 4 1 19 34 10.1177/1753465809352792 20051446

[B13] Vanhaesebroeck B Synthesis and function of 3-phosphorylated inositol lipids Annu Rev Biochem 2001 70 535 602 10.1146/annurev.biochem.70.1.535 11395417

[B14] Cantley LC The phosphoinositide 3-kinase pathway Science 2002 296 5573 1655 7 10.1126/science.296.5573.1655 12040186

[B15] Venable JD Phosphoinositide 3-kinase gamma (PI3Kgamma) inhibitors for the treatment of inflammation and autoimmune disease Recent Pat Inflamm Allergy Drug Discov 2010 4 1 1 15 10.2174/187221310789895603 20017720

[B16] Stoyanov B Cloning and characterization of a G protein-activated human phosphoinositide-3 kinase Science 1995 269 5224 690 3 10.1126/science.7624799 7624799

[B17] Kok K Regulation of p110delta PI 3-kinase gene expression PLoS One 2009 4 4 e5145. 19357769 10.1371/journal.pone.0005145 PMC2663053

[B18] Wymann MP Schneiter R Lipid signalling in disease Nat Rev Mol Cell Biol 2008 9 2 162 76 10.1038/nrm2335 18216772

[B19] Rommel C Camps M Ji H PI3K delta and PI3K gamma: partners in crime in inflammation in rheumatoid arthritis and beyond? Nat Rev Immunol 2007 7 3 191 201 10.1038/nri2036 17290298

[B20] Sadhu C Selective role of PI3K delta in neutrophil inflammatory responses Biochem Biophys Res Commun 2003 308 4 764 9 10.1016/S0006-291X(03)01480-3 12927784

[B21] Heit B PTEN functions to 'prioritize' chemotactic cues and prevent 'distraction' in migrating neutrophils Nat Immunol 2008 9 7 743 52 10.1038/ni.1623 18536720

[B22] Fukao T Koyasu S PI3K and negative regulation of TLR signaling Trends Immunol 2003 24 7 358 63 10.1016/S1471-4906(03)00139-X 12860525

[B23] Guha M Mackman N The phosphatidylinositol 3-kinase-Akt pathway limits lipopolysaccharide activation of signaling pathways and expression of inflammatory mediators in human monocytic cells J Biol Chem 2002 277 35 32124 32 10.1074/jbc.M203298200 12052830

[B24] Ishii KJ Potential role of phosphatidylinositol 3 kinase, rather than DNA-dependent protein kinase, in CpG DNA-induced immune activation J Exp Med 2002 196 2 269 74 10.1084/jem.20020773 12119352 PMC2193923

[B25] Ojaniemi M Phosphatidylinositol 3-kinase is involved in Toll-like receptor 4-mediated cytokine expression in mouse macrophages Eur J Immunol 2003 33 3 597 605 10.1002/eji.200323376 12616480

[B26] Seldon PM Suppression of lipopolysaccharide-induced tumor necrosis factor-alpha generation from human peripheral blood monocytes by inhibitors of phosphodiesterase 4: interaction with stimulants of adenylyl cyclase Mol Pharmacol 1995 48 4 747 57 7476903

[B27] Kirkham PA Cigarette smoke triggers macrophage adhesion and activation: role of lipid peroxidation products and scavenger receptor Free Radic Biol Med 2003 35 7 697 710 10.1016/S0891-5849(03)00390-3 14583334

[B28] Liang BT Galper JB Differential sensitivity of alpha o and alpha i to ADP-ribosylation by pertussis toxin in the intact cultured embryonic chick ventricular myocyte. Relationship to the role of G proteins in the coupling of muscarinic cholinergic receptors to inhibition of adenylate cyclase activity Biochem Pharmacol 1988 37 23 4549 55 10.1016/0006-2952(88)90671-5 3144283

[B29] Weingarten R Mastoparan interacts with the carboxyl terminus of the alpha subunit of Gi J Biol Chem 1990 265 19 11044 9 2113529

[B30] Williams HC Griendling KK NADPH oxidase inhibitors: new antihypertensive agents? J Cardiovasc Pharmacol 2007 50 1 9 16 10.1097/FJC.0b013e318063e820 17666910

[B31] Triantafilou M Triantafilou K Lipopolysaccharide recognition: CD14, TLRs and the LPS-activation cluster Trends Immunol 2002 23 6 301 4 10.1016/S1471-4906(02)02233-0 12072369

[B32] Katada T Synergistic activation of a family of phosphoinositide 3-kinase via G-protein coupled and tyrosine kinase-related receptors Chemistry and physics of lipids 1999 98 1-2 79. 10.1016/S0009-3084(99)00020-1 10358930

[B33] Niwa K Redox regulation of PI3K/Akt and p53 in bovine aortic endothelial cells exposed to hydrogen peroxide Antioxid Redox Signal 2003 5 6 713 22 10.1089/152308603770380016 14588144

[B34] Nishida M G alpha(i) and G alpha(o) are target proteins of reactive oxygen species Nature 2000 408 6811 492 5 10.1038/35044120 11100733

[B35] Shaw M Cohen P Alessi DR The activation of protein kinase B by H2O2 or heat shock is mediated by phosphoinositide 3-kinase and not by mitogen-activated protein kinase-activated protein kinase-2 Biochem J 1998 336 Pt 1 241 6 9806907 10.1042/bj3360241 PMC1219864

